# Neutrophil extracellular traps-targeting therapy with deoxyribonuclease 1 reduces large vessel occlusion-induced downstream microvascular thromboinflammation in a rat model of stroke

**DOI:** 10.1016/j.rpth.2025.103206

**Published:** 2025-10-06

**Authors:** Lucas Di Meglio, Mialitiana Solo Nomenjanahary, Laurine Bedoucha, Sebastien Dupont, Fatima Zemali, Véronique Ollivier, Clement Journe, Martine Jandrot-Perrus, Thomas Rambaud, Mikael Mazighi, Benoit Ho-Tin-Noe, Jean-Philippe Desilles

**Affiliations:** 1 Université Paris Cité, Faculté de Santé, Département de Pharmacologie, Optimisation Thérapeutique en Neuropsychopharmacologie, U1144 Institut National de la Santé et de la Recherche Médicale (INSERM), Paris, France; 2 Biological Resource Center and Department of Interventional Neuroradiology, Rothschild Foundation hospital, Paris, France; 3 Stroke-Link, French Clinical Research Infrastructure Network (F-CRIN), Paris, France; 4 Fédération Hospitalo-Universitaire (FHU) NeuroVasc, Paris, France; 5 Department of Neurology, Groupe Hospitalier Universitaire (GHU) Assistance Publique – Hôpitaux de Paris (AP-HP) Nord, Hôpital Lariboisière, Paris, France; 6 Université Paris Cité and Université Sorbonne Paris Nord, Département de Recherche Cardiovasculaire, INSERM U1148, Laboratory for Vascular Translational Science (LVTS), Paris, France; 7 Université Paris Cité, Département d’Imagerie Biomédicale, INSERM UMS34, Fédération de Recherche en Imagerie Multimodalité (FRIM), Paris, France

**Keywords:** deoxyribonuclease I, stroke, ischemic, neutrophils, blood-brain barrier, reperfusion injury, microcirculation

## Abstract

**Background:**

Neutrophil activation and neutrophil extracellular traps (NETs) participate in downstream microvascular thromboinflammation (DMT) and blood brain barrier disruption in acute ischemic stroke (AIS).

**Objectives:**

The aim of this study was to test whether deoxyribonuclease 1 (DNase 1) infusion, which cleaves extracellular DNA, could reduce DMT in a transient middle cerebral artery (MCA) occlusion stroke model in rats.

**Methods:**

Eighteen rats were subjected to 120-minute transient MCA occlusion. DNase 1 (3 mg/kg, 20% intravenous, and 80% intraperitoneal injection) or vehicle were randomly infused 30 minutes after MCA occlusion. Main outcome criteria were the infarct volume assessed with magnetic resonance imaging, neurological disability, and the rate of hemorrhagic transformation measured at 24 hours. Brain DMT was assessed with biomarkers of platelet, coagulation, and neutrophil activation quantified in brain homogenates.

**Results:**

The infusion of DNase 1 significantly reduced the infarct volume (*P* = .024) and improved 24-hour neurological outcome (P = .031) compared with vehicle. Staining for fibrin(ogen) and citrullinated histones H3 colocalized with extracellular DNA in the occluded microvessels. Brain thrombin–antithrombin complexes and fibrinogen deposits were significantly reduced in DNase 1-treated rats compared with vehicle (*P* = .027 and *P* = .036, respectively). The blood brain barrier disruption assessed with brain immunoglobulin G measurement and brain edema were also reduced in DNase 1-treated rats (*P* = .015 and *P* = .031, respectively).

**Conclusion:**

Our results confirm that NETs contribute to DMT during AIS and that early NET-targeting therapy may represent a new strategy to improve the clinical benefit of large vessel recanalization in AIS.

## Essentials

• Extracellular DNA worsens brain injury in stroke via downstream microvascular thrombosis (DMT).
• Rats underwent transient middle cerebral occlusion treated with deoxyribonuclease 1 (DNase 1) or vehicle.
• DNase 1 reduced brain damage and brain-blood barrier disruption by mitigating DMT.
• DNase 1 shows promise clinically by reducing DMT and improving thrombolysis.

## 1 Introduction

High admission neutrophil count [1,2] and neutrophil to lymphocyte ratio [2], and admission hyperglycemia [3], which primes neutrophil responsiveness to ischemia [4], were described as critical factors associated with unfavorable functional outcome in acute ischemic stroke (AIS) patients treated with endovascular therapy (EVT). Neutrophil extracellular traps (NETs), which are released upon neutrophil activation, have been shown to be positively correlated with AIS severity at onset and independently associated with post-AIS all-cause mortality [2,5]. NET-forming neutrophils were found throughout brain tissue of AIS patients, and elevated plasma NET biomarkers correlated with worse AIS outcomes [6,7].

Studies in animal models of AIS have further revealed that large vessel occlusion rapidly triggers a detrimental neutrophil response characterized by the formation of platelet/neutrophil aggregates occluding microvessels downstream of the occlusion site [[7], [8], [9], [10]]. NETs are cytotoxic and prothrombotic mediators, whose degradation by a preventive treatment with DNase 1 prior to experimental AIS onset in mice was shown to reduce infarct volume and improve functional outcome [7,11]. These results have thus indicated that NETs and extracellular DNA contribute to cerebral ischemia-reperfusion injury. In addition to these effects at the acute phase of experimental stroke, it was shown that NETs also impair beneficial vascular remodeling following permanent occlusion of the middle cerebral artery (MCA) [12]. However, it remains unclear how NETs mediate cerebral ischemia-reperfusion injury. In the present study, using a rat model of transient occlusion of MCA (tMCAO), we investigated whether DNase-1 administered early after MCA occlusion could help to reduce deleterious downstream microvascular thromboinflammation (DMT), and thus improve neurological outcome.

## 2 Methods

### 2.1 Data availability

The datasets generated during or analyzed during this study are not publicly available but are available from the corresponding author on reasonable request and with permission of all contributing authors. All experimental procedures were declared to the French Ministry of Research and authorized after ethics review, and all experiments were conducted in compliance with the Stroke Treatment Academic Industry Roundtable guidelines.

### 2.2 MCAO and reperfusion

**Figure 1:**
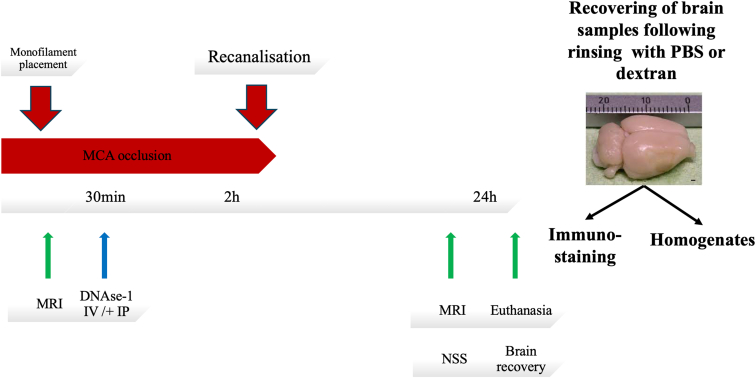
Experimental protocol. Initially, a monofilament was inserted, with subsequent MRI confirmation of MCA occlusion. DNAse-1 was administered 30 minutes after occlusion, utilizing both intravenous (20% via tail vein) and intraperitoneal (80%) routes. Recanalization was achieved 2 hours later by monofilament removal. At 24 hours, the protocol concluded with a second MRI, neurological severity scoring (NSS), and euthanasia, followed by the recovery of brain samples for further analysis. Before brain extraction, tissues were preemptively rinsed with phosphate buffer saline (PBS) or dextran by intracardiac infusion. The collected tissues were then processed into homogenates for biochemical assessments, while a selected slice underwent immunostaining. MCA, middle cerebral artery; MRI, magnetic resonance imaging.

Animal experiments are reported in accordance with the ARRIVE guideline (Animal Research: Reporting of In Vivo Experiments). Male Sprague–Dawley rats (330–400 g; Janvier) were subjected to 120 minutes of transient monofilament (4041PK10, Doccol) tMCAO, as described [13]. After 30 minutes of occlusion, 20% of the DNase 1 (Dornase Alfa, Pulmozyme, Roche) or vehicle was randomly administered intravenously through the tail vein, and 80% intraperitoneally (total dose = 3 mg/kg, 1 mL) [11]. Computer-based randomization was used to allocate drug regimens infused to each animal. Experiments were blinded, and the operator was unaware of group allocation during surgery and outcome assessment (Figure 1). To confirm the *in vivo* biological activity of DNase at the dosage used, as previously reported [11], we performed an additional series of experiments in mice using the same treatment protocol (see Supplementary Methods).

### 2.3 Magnetic resonance imaging

All magnetic resonance imaging (MRI) were performed on a 7-Tesla small animal scanner (Bruker Pharmascan) equipped with the Paravision 6.0.1 software platform (Bruker). MRI was performed with a planar surface coil (30 mm inner diameter) under 1.5% isoflurane anesthesia. Animals were placed prone onto an imaging cradle, their temperature was maintained at 37 °C, and their respiration was monitored continuously. The MRI protocol included 3-dimensional time-of-flight angiography (T1-weighted fast low-angle shot images; TE 2.5 ms, TR 15 ms, matrix 256 × 256 × 128, flip angle 20°, field of view 37 × 37 × 30 mm); 2-dimensional diffusion-weighted imaging (spin—echo with echo—planar imaging readout; TE 34 ms, TR 2300 ms, matrix 128 × 128, field of view 33 × 33 mm, 17 slices, slice thickness 0.8 mm with no gap, 6 directions, b values 0, 500, and 1000 s/mm^2^); and apparent diffusion coefficient map was calculated with all the b values. A first MRI was performed immediately after MCAO to confirm the absence of flow in the right MCA (Figure 1). The second MRI was performed at 24 hours and included 2 additional sequences: susceptibility weighted imaging ([SWI], flow compensated fast low-angle shot images; TE 18 ms, TR 597.411 ms, matrix 256 × 256, flip angle 40°, 3 averages, field of view 33 × 33 mm, 17 slices, slice thickness 0.8 mm) and T2 weighted rapid acquisition with relaxation enhancement imaging (TE 33 ms, TR 2700 ms, Rare factor 8, matrix 256 × 256, field of view 33 × 33 mm, 17 slices, slice thickness 0.8 mm).

### 2.4 Animal sample size calculation

The study was designed to detect a relative 50% difference in cerebral infarct volume between groups (DNase 1 vs vehicle), with 90% power and a 5% α-level, assuming a mean±SD infarct volume of 380±120 mm^3^ in the vehicle group, based on previous experiments. It was thus calculated that 9 rats would be sufficient in each group. Two additional animals were included in each group to anticipate exclusions because of procedural complications (tMCAO failure or anesthetic-related mortality).

### 2.5 Exclusion criteria

Animals that met one of the following predefined criteria were excluded from the analysis: subarachnoid hemorrhage, absence of confirmed MCAO on the first MRI, failure to administer the treatment through the vein, death because of anesthetic overdose, and absence of recanalization.

### 2.6 Determination of infarct volume, hemorrhagic transformation, and neurological deficit

The neurological deficit was evaluated using a modified neurological severity score, which is a composite of motor, sensory, and balance tests [14]. Infarct volume and edema calculation were performed blindly with Horos software on 17 slices of T2 sequences. Edema was expressed as the total volume of the infarcted hemisphere divided by the volume of both hemispheres. Hemorrhagic transformation was assessed on the SWI sequence at 24 hours as a dichotomic variable.

### 2.7 Blood brain barrier disruption and microvascular thromboinflammation

For measurements in brain homogenates, the brains were recovered after intracardiac perfusion with 40 mL of saline. The brains were then sliced into 2 parts: the right hemisphere and the left hemisphere. Each hemisphere was crushed and centrifuged with 1× phosphate buffer saline (PBS) and 1% protease inhibitor to extract proteins. The supernatants were then stored at −20 °C. ELISA-based methods for rat immunoglobulin G (IgG) (Abcam; IgG rat ELISA kit; ab157737), thrombin–antithrombin (Enzygnost, Siemens, OWMG15), fibrinogen (Molecular Innovation, RFBGNKT-216), myeloperoxidase (MPO, Hycult Biotech, HK105), and matrix-metalloproteinase 9 (R&D Systems, RMP900) were used to quantitatively measure blood brain barrier (BBB) disruption and microvascular thromboinflammation. Values are expressed as ng reported to the total protein count of the corresponding hemisphere. One rat from the DNase 1-treated group was excluded from the ELISA analysis because no saline perfusion could be performed at the time of euthanasia.

### 2.8 Immunohistochemistry

Two Supplemental rats were subjected to 120-minute tMCAO. They were euthanized at 6 hours after recanalization, and brains were recovered after intracardiac perfusion of saline (40 mL) with dextran (Sigma Aldrich). Slices at +0.70 mm posterior to bregma were fixed in 3.7% paraformaldehyde and embedded in paraffin. After deparaffinization, tissue sections were permeabilized, washed, and incubated with primary antibodies to fibrinogen (10 μg/mL, Dako, A0080), myeloperoxidase (10 μg/mL, Dako, A0398), and H4 citrullinated 3 (1/200, Merck Millipore, 07-596), followed by incubation with fluorescent secondary antibodies (1/400, Biotinylated goat antirabbit, Invitrogen, B2770) and streptavidin conjugated to Alexa Fluor 647 (10 μg/mL, Invitrogen, S32357). Tissue sections were finally counterstained with Hoechst 33342 (10 μg/mL, Life Technologies) and mounted in fluorescent mounting medium (Dako).

The acquisition was performed on a Leica DMI8 microscope, and pictures were analyzed with LAS X software (Leica Microsystems).

### 2.9 Statistical analysis

Data were analyzed using a nonparametric analysis of variance (Kruskal–Wallis), followed by the Wilcoxon rank-sum test for comparison of paired data or by the Mann Whitney *U*-test for comparison of unpaired data. Results are presented as median±IQR and percentage for qualitative variables. For statistical analysis, PrismGraph 4.0 software (GraphPad Software) was used. Values of *P* < .05 were considered statistically significant.

## 3 Results

### 3.1 DNase 1 infusion after MCAO reduces final infarct volume and BBB disruption

**Figure 2:**
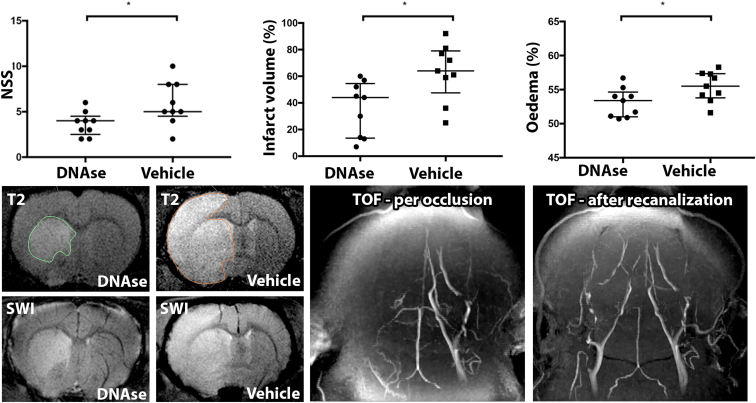
Upper panel. Effect of deoxyribonuclease (DNase) 1 treatment on 24 hours clinical outcome, infarct volume, and brain edema. DNase 1-treated rats have a significant reduction of their neurological severity score (NSS) score, infarct volume, and edema compared with vehicle-treated rats. On the lower panel, representative MRI-images of the 24 hours-infarct volume (T2), the absence of hemorrhagic transformation assessed thanks to SWI, the confirmation of the occlusion and the recanalization of the MCA (TOF). Infarct volume in 10^-3^mm^3^. Edema is express as the % of the infarcted hemisphere reported to the volume of both hemispheres. MCA, middle cerebral artery; MRI, magnetic resonance imaging; SWI, susceptibility weighted imaging; TOF, time-of-flight. ^a^*P* < .05.

Twenty-two rats were randomized to receive either DNase-1 or vehicle. During surgery, 4 rats were excluded from the study: 2 from each group. The exclusions were due to the following reasons: 2 rats showed an absence of occlusion on MRI, indicating a failure of monofilament placement; 1 rat died during the first anesthesia; and 1 rat experienced persistent occlusion for 24 hours because the monofilament was not removed. DNase 1-treated rats had a reduced infarct volume at 24 hours compared with vehicle-treated rats (median [IQR] 262 [109-289] 10^-3^mm^3^ vs 351 [324-383] 10^-3^mm^3^; *P* = .024; Figure 2). This result was consistent with the better neurological outcome of DNase 1-treated rats compared with vehicle-treated rats (4 [3-4] vs 5 [5-8]; *P* = .031; Figure 2). No hemorrhagic transformation was observed in either groups. BBB disruption as assessed by the measurement of brain IgG content was also reduced in ischemic hemispheres of DNase 1-treated rats (20 [13-26] vs 37 [30-80] ng/ng of protein; *P* = .015). This reduction in brain IgG content of DNase 1-treated rats was consistent with a reduction of brain edema compared with vehicle-treated rats (51 [51-53] vs 56 [54-56] % of total brain volume; *P* = .031; Figure 2).

### 3.2 Presence of fibrin(ogen) deposits and NETs in rats ischemic brain microvasculature

**Figure 3:**
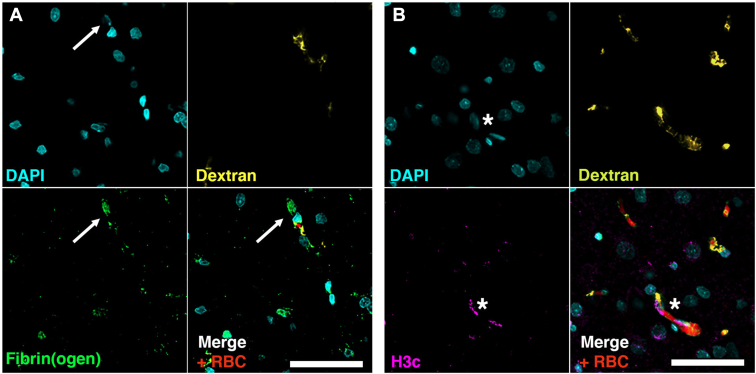
Representative images of the presence of NETs and fibrin(ogen) deposits in brain microvasculature of ischemic hemispheres (superficial MCA territory). (A) Staining of fibrin(ogen) (green) reveal colocalization of fibrin(ogen) deposits and extracellular DNA (DAPI, blue) in occluded microvessels (white arrow). These microvessels exhibit stagnation of dextran (yellow) perfused before euthanization and red blood cells (red, autofluorescence). (B) Presence of citrullinated histones (H3c, purple) positive extracellular DNA (Dapi, blue) in occluded microvessels (white star) suggesting of the presence of NETs. These microvessels exhibit the same stagnation of dextran (yellow) perfused before euthanization and red blood cells (red, autofluorescence). MCA, middle cerebral artery; NET, neutrophil extracellular traps.

In ischemic brain areas, occluded microvessels could be located by the stagnation of dextran and red blood cells (Figure 3). Fibrin(ogen) staining colocalized with extracellular DNA in these occluded microvessels (Figure 3A). Citrullinated histones H3 staining colocalized with extracellular DNA suggesting the presence of NETs in these occluded microvessels (Figure 3B). Nonischemic brain areas did not exhibit stagnation of dextran or red blood cells, nor fibrin(ogen) and extracellular DNA deposits (data not shown). In mice, DNase 1 treatment significantly reduced the number of NETs in ischemic hemispheres of DNase 1-treated mice compared with vehicle-treated mice (median 22 [IQR 18-40] NETs vs vehicle 72 [IQR 59-89] NETs; *P* = .041). However, this reduction was not associated with early neurologic improvement, as neurologic severity scores at 24 hours were similar between groups (7 [IQR 6-8] in both the DNase1-treated and the vehicle groups), nor a reduction of neuronal death assessed by Fluoro–Jade staining, see Supplementary Results and Figure.

### 3.3 DNase 1 infusion is associated with a reduced DMT response to ischemia

**Figure 4:**
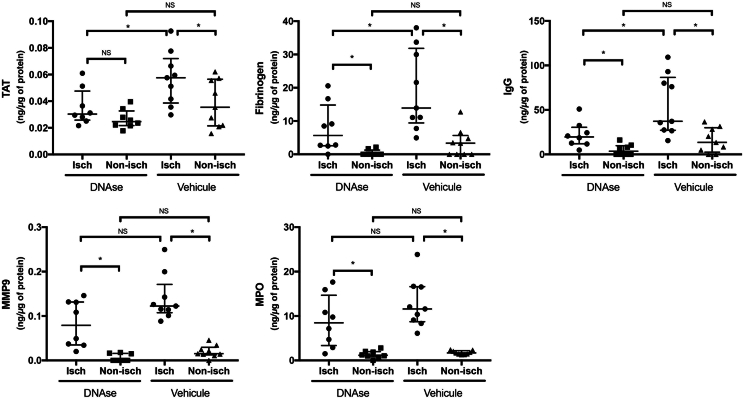
Effect of deoxyribonuclease (DNase) 1 treatment on brain thromboinflammation biomarkers. In both groups, all thromboinflammation biomarkers were significantly increased in the ischemic hemisphere compared with the nonischemic hemisphere. DNase 1 treatment was associated with a significant (∗) reduction of TAT, fibrinogen, and IgG in the ischemic hemisphere compared with vehicle. MPO and MMP-9 were also decreased in ischemic hemisphere of DNase 1-treated rats compared with vehicle without reaching significance (NS). TAT, thrombin/antithrombin; IgG, immunoglobulin G; MMP, matrix-metalloproteinase; MPO, myeloperoxidase. Values are expressed as medians+/-interquartile range.

Biomarkers of DMT, brain thrombin–antithrombin complexes and fibrinogen deposits, were significantly lower in ischemic hemispheres of DNase 1-treated rats compared with vehicle-treated rats (30 [28-40] vs 58 [44-66] 10^-3^ng/ng of protein; *P* = .027 and 6 [3-11] vs 14 [11-30] ng/mg of protein; *P* = .036, respectively). On the other hand, markers of neutrophil and glial activation, MPO and matrix-metalloproteinase 9 were lower in ischemic hemispheres of DNase 1-treated rats compared with vehicle without reaching significance (9 [4-12] vs 12 [9-17] ng/ng of protein; *P* = .200 and 79 [37-131] vs 122 [114-143] 10^-3^ng/ng of protein; *P* = .167, respectively; Figure 4).

## 4 Discussion

Here, using a tMCAO stroke model in rats, we show that treatment with DNase-1 after MCA occlusion is associated with a 24-hour favorable outcome and a reduced infarct volume compared to vehicle. We further show that the microvessels of ischemic hemispheres are occluded with fibrin(ogen) deposits and NETs and that DNase-1 administration is associated with a reduction of this DMT and BBB disruption.

Our results are in accordance with those of De Meyer et al. [11] and Denorme et al. [7]. They showed in a mouse model of tMCAO that levels of circulating nucleosomes and DNA were increased after AIS. Furthermore, preventive DNase-1 treatment significantly improved stroke outcome in these studies. Peña-Martínez et al. [15] also found that NETs have a deleterious effect during AIS, which can be mitigated by DNase-1. Our study provides new mechanistic insight that after occlusion DNase-1 treatment reduces DMT as shown by the diminution of thrombin–antithrombin and fibrinogen accumulation in the brain of DNase-1-treated rats. Considering that various immunostimulatory effects of purified chromatin components (ie, double stranded DNA and histones) have been previously demonstrated, both *in vitro* and *in* vivo [16,17], it is worth noting that DNase-1-generated DNA degradation products do not cause inflammatory complications.

DMT in tMCAO is initiated immediately after occlusion and propagated through the venous compartment in close association with marginating leukocytes [9,10]. NETs have been shown to foster thrombosis in various models by inducing activation of platelets [18] and endothelial cells [19], inhibiting activated protein C-mediated negative feedback of the coagulation cascade [20], and by directly supporting thrombin activation [21]. While, NETs have been shown to form in the lumen of the brain microvasculature [22], Pena-Martinez et al. [23] exhibited that they do so in a platelet-toll-like receptor 4 dependent way. Through its ability to cleave NETs, our data suggest that DNase-1 reduces the NETs-associated thrombosis in the brain microvasculature and therefore improves stroke outcome. Importantly, an upstream strategy targeting NET formation itself has also been shown to yield benefits in AIS treatment, as evidenced by the work of Denorme et al. [7]. This highlights the therapeutic potential of addressing both the formation and the presence of NETs in AIS setting. Furthermore, extracellular histones, which are contained in NETs, have been shown to be highly cytotoxic to endothelial cells [24]. Intravascular fibrin(ogen) accumulation and sites of leukocyte adhesion in venules and arterioles have also been associated with BBB disruption [10]. Although speculative, the reduction in BBB disruption by DNase-1 observed in this study could therefore be secondary to either the reduction in DMT or to the clearance of cytotoxic histones after NETs degradation.

This study has several limitations. First, our initial immunohistochemical analysis was limited to 2 rats, as it was originally intended only to illustrate the presence of NETs and fibrin(ogen) deposits in ischemic microvasculature. To strengthen the robustness of this finding, we subsequently replicated the experiment in a mouse model with a larger sample size and blinded quantification. Second, the complexity of the protocol—including DNase administration, transient MCA occlusion, and serial MRI—did not allow for concurrent plasma collection. Despite repeated attempts, technical and sensitivity limitations prevented reliable quantification of circulating NET markers such as cell-free DNA, nucleosomes, MPO, or elastase. We therefore cannot directly demonstrate NET degradation in plasma, although previous studies have confirmed the efficacy of the DNase dose used [7,11]. In addition, in the complementary mouse experiments, DNase1 reduced NET burden but did not improve neurological severity scores or neuronal degeneration assessed by Fluoro–Jade, in contrast with the beneficial effects observed in rats. These discordant results highlight the importance of the experimental model and outcome measures, and we now acknowledge them as a limitation of our study. Finally, the generalizability of our findings is limited by the exclusive use of male animals and the absence of long-term behavioral outcome assessment.

To be noted, there are indications that NETs in human stroke thrombi contribute both to the thrombus extracellular scaffold and to its resistance to tPA-mediated thrombolysis [[25], [26], [27]]. DNase-1 has been shown to accelerate *ex vivo* t-PA-induced thrombolysis of these thrombi [25,26]. DNase-1 therefore exhibits a dual interest for AIS treatment as it has been shown to both reduce DMT and potentially improve proximal recanalization. There are currently 2 ongoing clinical trials assessing the safety and efficacy of IV DNase 1 infusion (Dornase Alfa, Pulmozyme, Roche) in AIS patients on top of IV tPA and EVT (EXTEND-IA DNase, NCT05203224 and NETs-target, NCT04785066).

This study strengthens the data that DNase-1 offers a reliable therapeutic strategy to limit injury resulting from AIS in association with IV tPA and EVT.
