# Supplementary material for: Neutrophil extracellular traps-targeting therapy with deoxyribonuclease 1 reduces large vessel occlusion-induced downstream microvascular thromboinflammation in a rat model of stroke
Source: Res Pract Thromb Haemost. 2025 Oct 6;9(7):103206. doi: 10.1016/j.rpth.2025.103206 (PMC12603741; doi:10.1016/j.rpth.2025.103206)
Supplement: Supplementary Material [file mmc1.docx]

**Supplemental Materials and Methods**

**Animal Model**

Male C57BL/6 mice (8 to 12 weeks old) were used in this study. Animals were housed under standard laboratory conditions with ad libitum access to food and water. All experimental protocols were approved by the local institutional ethics committee.

**Ischemic Stroke Model (tMCAO)**

Transient middle cerebral artery occlusion (tMCAO) was induced by inserting a standardized monofilament (Doccol Corp., Redlands, CA) into the right internal carotid artery to block the origin of the right middle cerebral artery. The filament remained in place for 60 minutes to achieve ischemia. Anesthesia was induced with 5% isoflurane and maintained at 2% via inhalation. Buprenorphine was administered one hour before surgery and every 6 hours thereafter as needed for analgesia.

One mouse died during surgery and was excluded from the final analysis.

**Occlusion Protocol and DNase Administration**

Thirty minutes after occlusion onset, mice received either recombinant human DNAse 1 (Pulmozyme, 3 mg/kg) or an equivalent volume of saline. The dose was administered as 80% intraperitoneally (IP) and 20% intravenously (IV) via retro-orbital injection.

**Tissue Collection and Immunohistochemistry**

Mice were euthanized 24 hours after treatment. Brains were harvested, divided into three coronal blocks, and fixed in zinc-based fixative for 24 hours. Tissues were then dehydrated, embedded in paraffin, and 10-μm-thick sections were cut from the second block for histological analysis.

Neutrophil extracellular traps (NETs) were detected by dual immunofluorescence staining using antibodies against citrullinated histone H3 (cit-H3, ab281584) and Ly6G (BP0075-1, Invivo). Nuclear staining was performed with DAPI to visualize DNA. Sections were incubated with primary antibodies followed by fluorophore-conjugated secondary antibodies.

**Quantification of NETs**

Quantification was performed on the ischemic hemisphere using a LEICA fluorescence microscope. NETs were defined as extracellular filamentous structures double-positive for cit-H3 and Ly6G. Results are expressed as the number of NETs per hemisphere.

All image analyses were performed blinded to treatment by two independent observers.

**FluoroJade Staining and infarct surface measurement**

Brain sections were obtained from zinc-fixed tissue and deparaffinized by successive 2-minute incubations in xylene (×2), xylene/100% ethanol (1:1), 95%, 70%, and 50% ethanol, followed by distilled water. Slides were then incubated in 1% NaOH/80% ethanol (20 mL of 5% NaOH + 80 mL absolute ethanol) for 5 minutes, rinsed in 70% ethanol and distilled water (2 minutes each), and immersed in 0.06% KMnO₄ for 10 minutes with gentle agitation (50 rpm), followed by distilled water (2 minutes). Fluoro-Jade B staining was performed at a final concentration of 0.0004% (prepared by diluting 4 mL of a 0.01% stock solution in 100 mL of 0.1% acetic acid) for 20 minutes in the dark under agitation. After rinsing (3 × 1 minute in distilled water), slides were counterstained with DAPI (1:1000, 5 minutes, dark), rinsed again (3 × 1 minute), dried at 50 °C for 10 minutes, cleared in xylene (1 minute), and mounted with EUKITT®. Images were acquired using a Leica fluorescence microscope, with excitation of Fluoro-Jade B signal by a green laser. Planimetric measurements (Image J software, National Institutes of Health, Bethesda, MD) were performed blinded to the treatment group and were used to calculate infarct surface as previously published (DOI:10.1161/STROKEAHA.115.010721).

**Supplemental Results**

**Quantitative Analysis of NETs in Ischemic Brain Tissue**

To confirm the presence of neutrophil extracellular traps (NETs) and evaluate the effect of DNAse 1 treatment on their formation, we performed immunofluorescence analysis on brain sections from a separate cohort of mice subjected to transient middle cerebral artery occlusion (tMCAO). A total of 12 mice were included (n=6 per group, DNAse 1 vs. vehicle).

NETs were identified as extracellular filamentous structures positive for both citrullinated histone H3 (cit-H3) and Ly6G, with colocalization confirmed by fluorescence microscopy. Quantification was performed in the ischemic hemisphere using blinded analysis by two independent observers.

Mice treated with DNAse 1 exhibited a significant reduction in the number of NETs compared to vehicle-treated mice (median 22 [IQR 18–40] NETs versus vehicle 72 [IQR 59–89] NETs, p = 0.041). These findings support the in vivo efficacy of DNAse 1 in degrading NETs following ischemic stroke. However, this reduction was not associated with early neurological improvement, as neurological severity scores at 24 hours were similar between groups (7 [IQR 6–8] in both DNase1-treated and vehicle groups, Supplemental Figure 2) nor a reduction of infarcts surface assessed by FluoroJade staining (51 [IQR 44-57] vs 55 [IQR 45-65] cm2, Supplemental Figure 3).

A representative image and quantification graph are presented in Supplemental Figure 1 and 2.


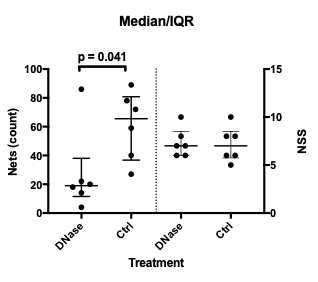

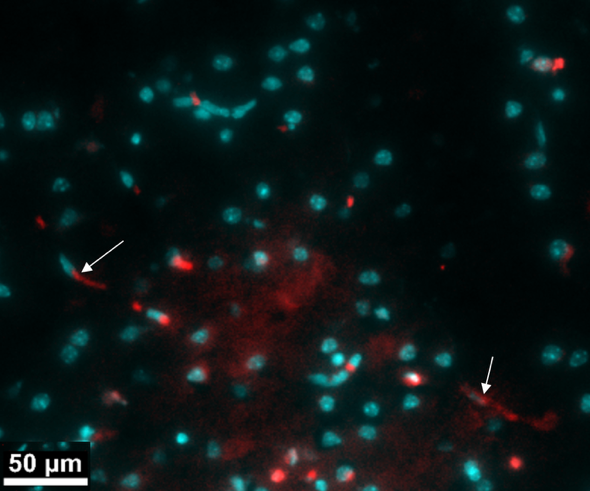


**Supplemental Figure 1. DNase treatment reduces NETs formation in vivo.**

Quantification of NETs (neutrophil extracellular traps) in brain sections of mice treated with DNase or vehicle (Ctrl). **Left**: each dot represents one animal. Data are shown as median and interquartile range (IQR). DNase significantly reduced NET counts compared to control (median 22 [14–27] vs. 72 [49–82]; *p* = 0.041). Neurological severity scores at 24 hours were similar between groups (7 [IQR 6–8] in both DNase1-treated and vehicle groups **Right**: representative immunofluorescence image of brain parenchyma showing NETs (arrows) as elongated structures positive for both DNA (blue) and H3Cit staining (red).


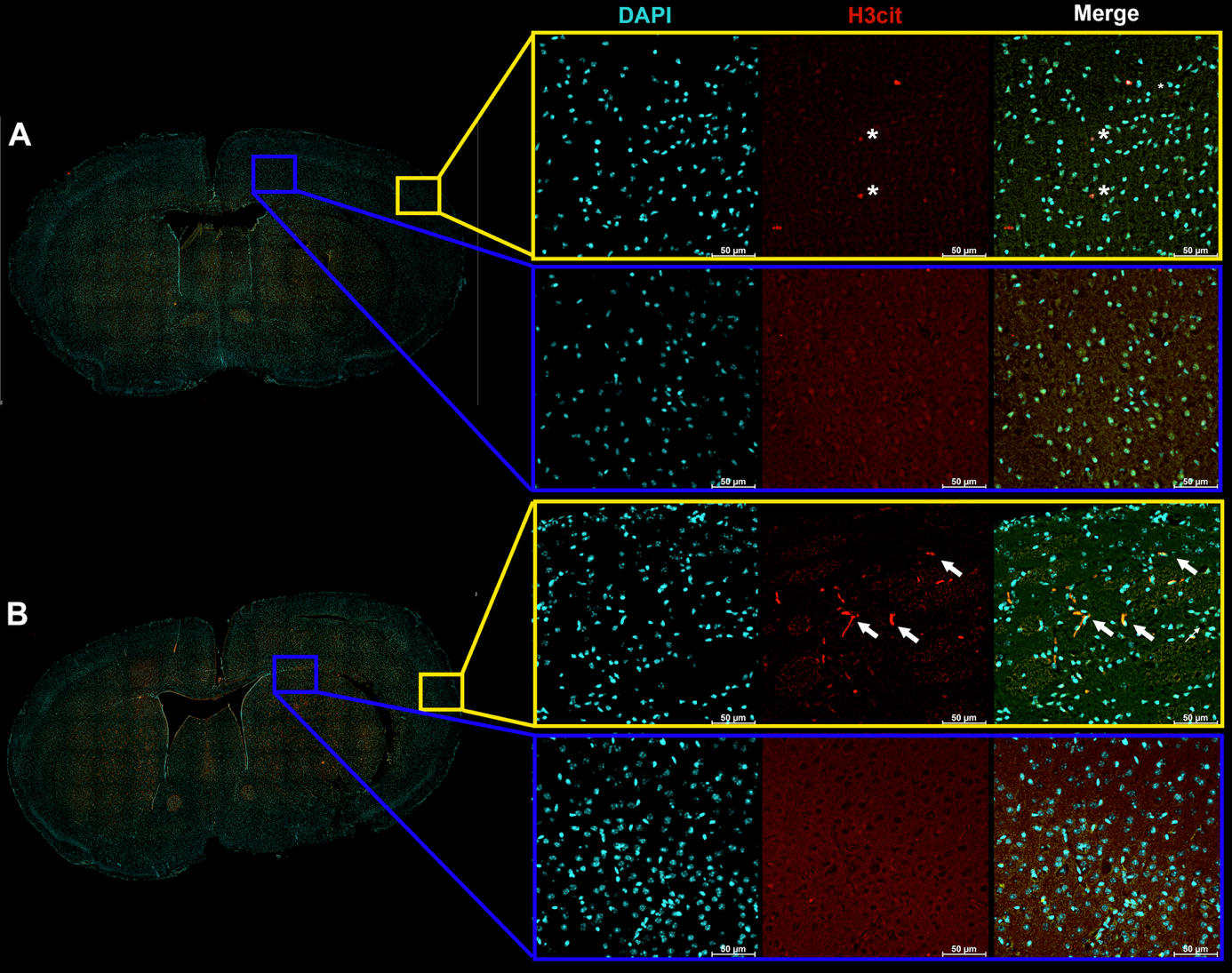


**Supplemental Figure 2. Representative immunostaining of brain sections from DNase1-treated (A) and vehicle-treated (B) mice at 24 hours post-stroke.** Whole-brain sections were stained for DNA (DAPI, blue) and citrullinated histone H3 (H3cit, red). Insets show higher magnification of the ischemic core in the MCA territory (yellow boxes) and of the unaffected homolateral ACA territory (blue boxes). Asterisks (*) indicate H3cit-positive cells with round nuclei, consistent with incomplete chromatin decondensation. White arrows point to decondensed DNA structures positive for H3cit, suggestive of neutrophil extracellular traps (NETs). Scale bars: 50 μm.


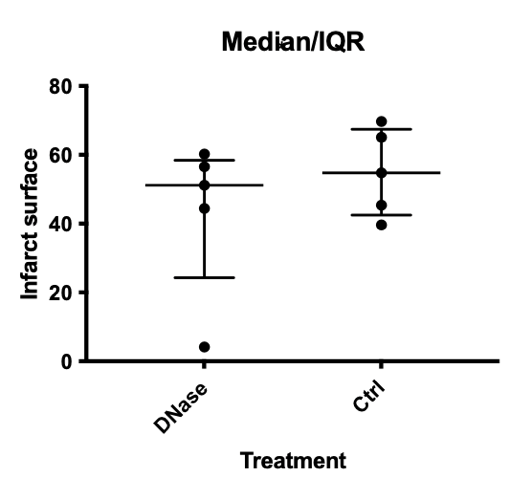

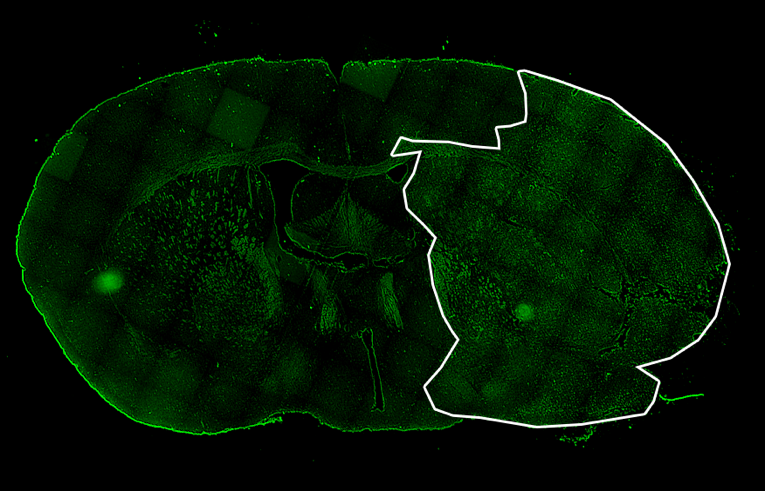


**Quantification of infarcted area using Fluoro-Jade staining in mouse brains.**

Representative coronal brain section stained with Fluoro-Jade at 24 hours after tMCAO, showing the delineated infarcted area (white outline, right panel). Quantification of infarct surface did not reveal significant differences between DNase1-treated and vehicle-treated groups (left panel, data expressed as median and interquartile range).
